# Detailed Phenotype of GLA Variants Identified by the Nationwide Neurological Screening of Stroke Patients in the Czech Republic

**DOI:** 10.3390/jcm10163543

**Published:** 2021-08-12

**Authors:** Petra Reková, Gabriela Dostálová, David Kemlink, Jaroslava Paulasová Schwabová, Zora Dubská, Manuela Vaneckova, Martin Mašek, Ondřej Kodet, Helena Poupětová, Stella Mazurová, Aneta Rajdova, Eva Vlckova, Alena Táboříková, Štěpánka Fafejtová, Miroslava Nevsimalova, Aleš Linhart, Aleš Tomek

**Affiliations:** 1 Department of Neurology and Centre of Clinical Neuroscience, First Faculty of Medicine, Charles University and General University Hospital in Prague, 128 08 Prague, Czech Republic; petra.rekova@vfn.cz; 2 2nd Department of Medicine—Department of Cardiovascular Medicine, First Faculty of Medicine, Charles University, 128 08 Prague, Czech Republic; gabriela.dostalova@vfn.cz (G.D.); ales.linhart@vfn.cz (A.L.); 3 Department of Neurology, Second Faculty of Medicine, Charles University and Motol University Hospital in Prague, 150 06 Prague, Czech Republic; jaroslava.schwabova@fnmotol.cz (J.P.S.); ales.tomek@fnmotol.cz (A.T.); 4 Department of Paediatric Neurology, Second Faculty of Medicine, Charles University and Motol University Hospital in Prague, 150 06 Prague, Czech Republic; 5 Department of Ophthalmology, First Faculty of Medicine, Charles University and General University Hospital in Prague, 128 08 Prague, Czech Republic; zora.dubska@vfn.cz; 6 Department of Radiology, First Faculty of Medicine, Charles University and General University Hospital in Prague, 128 08 Prague, Czech Republic; manuela.vaneckova@vfn.cz (M.V.); martin.masek@vfn.cz (M.M.); 7 Department of Dermatovenerology, First Faculty of Medicine, Charles University and General University Hospital in Prague, 128 08 Prague, Czech Republic; ondrej.kodet@vfn.cz; 8 Biotechnology and Biomedicine Centre, Academy of Science, Charles University, 252 50 Vestec, Czech Republic; 9 Institute of Anatomy First Faculty of Medicine, Charles University in Prague, 128 08 Prague, Czech Republic; 10 Department of Paediatrics and Inherited Metabolic Disorders, First Faculty of Medicine, University and General University Hospital in Prague, 128 08 Prague, Czech Republic; helena.poupetova@vfn.cz (H.P.); stella.mazurova@vfn.cz (S.M.); 11 Department of Neurology, Faculty of Medicine, Masaryk University and University Hospital Brno, 625 00 Brno, Czech Republic; rajdova.aneta@fnbrno.cz (A.R.); vlckova.eva@fnbrno.cz (E.V.); 12 Department of Neurology and Stroke Centre, Country Hospital Chomutov, 430 12 Chomutov, Czech Republic; alena.taborikova@kzcr.eu; 13 Department of Neurology and Stroke Centre, Hospital Karlovy Vary, 360 01 Karlovy Vary, Czech Republic; stepanka.fafejtova@seznam.cz; 14 Department of Neurology, Hospital Ceske Budejovice, 370 01 České Budějovice, Czech Republic; nevsimalova.miroslava@nemcb.cz

**Keywords:** Fabry disease, GLA gene variants, phenotype, stroke, screening programs, data sharing

## Abstract

Fabry disease (FD) is a rare X-linked disorder of glycosphingolipid metabolism caused by pathogenic variants within the alpha-galactosidase A (GLA) gene, often leading to neurological manifestations including stroke. Multiple screening programs seeking GLA variants among stroke survivors lacked detailed phenotype description, making the interpretation of the detected variant’s pathogenicity difficult. Here, we describe detailed clinical characteristics of GLA variant carriers identified by a nationwide stroke screening program in the Czech Republic. A total of 23 individuals with 8 different GLA variants were included in the study. A comprehensive diagnostic workup was performed by a team of FD specialists. The investigation led to the suggestion of phenotype reclassification for the G325S mutation from late-onset to classical. A novel variant R30K was found and was classified as a variant of unknown significance (VUS). The typical manifestation in our FD patients was a stroke occurring in the posterior circulation with an accompanying pathological finding in the cerebrospinal fluid. Moreover, we confirmed that cornea verticillata is typically associated with classical variants. Our findings underline the importance of detailed phenotype description and data sharing in the correct identification of pathogenicity of gene variants detected by high-risk-population screening programs.

## 1. Introduction

Fabry disease (FD) is a rare inherited X-linked monogenic disorder (OMIM #301500). Variants in the GLA gene result in altered or missing production of a lysosomal enzyme alpha-galactosidase A (α-Gal A; EC 3.2.1.22) [1,2]. Consequently, they lead to an inability to properly hydrolyse the terminal α-galactose moieties, disrupting the glycosphingolipids catabolic pathway and leading to the accumulation of substrates, predominantly globotriaosylceramide, Gb3 [3].

The disease phenotypes are heterogeneous, scaling from severe to asymptomatic cases. The classical phenotype is characterised by multiorgan involvement. Typical manifestations occurring early in life include neuropathic pain, hypohidrosis, gastrointestinal symptoms, development of angiokeratomas and cornea verticillata. The life expectancy of FD patients is limited by cardiac damage, progressive kidney function deterioration or central nervous system involvement [2]. A large group of patients suffer from milder forms of the disease with late-onset phenotypes. The group consists of patients with variously expressed organ manifestations, sometimes limited to one organ, often to the heart [4,5].

At a biochemical level, severely decreased or absent α-GAL A activity and increased level of globotriaosylsphingosine, lyso-Gb3 are hallmarks in males with a classical phenotype [6,7]. The reduction of enzymatic activity and lyso-Gb3 increase is less pronounced in females and later-onset variants. In general, heterozygous women are less affected than hemizygous male patients, in some cases remaining asymptomatic possibly due to skewed X-chromosome inactivation [8].

The classical phenotype incidence of FD is estimated at 1 in 25,000 to 1 in 40,000; the nonclassical seems to be about 10-fold more frequent [9]. The availability of specific therapy and simplified diagnostic methods represented mainly by dry blood spot (DBS) technique testing [10] led to increased efforts to diagnose FD, including newborn [11] and high-risk populations screening programs [12]. 

A significant increase in the gene variants discovered by the screening has refined FD incidence estimations. Unfortunately, clinical characteristics of detected variants are often missing in the literature. Thus, some variants have been misclassified in terms of pathogenicity [12]. Misclassifications as pathogenic have a psychological impact on patients and their families, administration of unnecessary treatments and can influence research and clinical trials, thus leading to wasting healthcare resources. In contrast, false classification of a variant as benign could disqualify patients from accessing effective therapy. Therefore, it is important to share patients’ data, enabling the integration of genetic and clinical information to avoid variant misinterpretations with the necessity of later reclassification.

Here, we describe detailed clinical phenotype characteristics in 16 individuals with GLA gene variants found in our nationwide FD screening study in stroke patients and their 7 relatives.

## 2. Materials and Methods

### 2.1. Study Design and Patient Selection

We performed a prospective nationwide multicentre study including consecutive stroke patients admitted during a selected period of 3 months in 35 stroke centres in the Czech Republic.

A total of 986 consenting patients presenting with an acute cerebrovascular disease admitted during March 2018, October 2018 and March 2019 were included in the study irrespective of stroke subtype. We included patients presenting during the study duration with a transient ischemic attack, ischemic stroke, intracerebral haemorrhage, subarachnoid haemorrhage and cerebral venous thrombosis, regardless of stroke aetiology or patient age. There were no other exclusion or inclusion criteria. All patients gave written informed consent to the study. FD was diagnosed using DBS in a stepwise manner: in males, enzymatic activity, lyso-Gb3 quantification, if positive, followed by GLA gene sequencing; and in females, GLA sequencing, followed by lyso-Gb3. The study has led to the identification of 16 index cases with a GLA gene variant. Pedigree analysis and subsequent cascade family genetic testing were performed in all cases except for D313Y variant carriers, leading to the identification of 7 relatives carrying a gene variant. Altogether, these 23 cases represent the current study population. A comprehensive diagnostic workup of all cases to determine FD organ manifestations was performed by a team of FD experts from the Czech National FD centre. All examinations were performed according to the internal protocol of the Czech FD centre for initial disease evaluation, which reflects international recommendations [13].

### 2.2. FD Organ Manifestation Assessment

#### 2.2.1. Clinical Assessment

Medical history focused on FD-related signs and symptoms was obtained from all study participants. Basic clinical examination, including blood pressure, heart rate, respiratory rate, height and weight measurements, were followed by specialised clinical examinations.

#### 2.2.2. Nervous System Assessment

Structured clinical examination with an emphasis on the presence of typical neurological symptoms of FD was carried out. Recorded symptoms included signs of peripheral neuropathy, gastrointestinal symptoms, changes in sweating, heat and exercise tolerance and cerebrovascular events. To evaluate white matter lesions (WMLs), we performed non-contrast brain magnetic resonance imaging (MRI) using a 3T MRI scanner (MAGNETOM Skyra, Siemens Healthcare, Erlangen, Germany). The MRI protocol comprised of T1W, T2W, FLAIR, DWI and SWI sequences. The occurrences of WMLs were rated according to Fazekas scale [14]. Cerebral blood vessels ultrasound and/or computed tomography or magnetic resonance angiography was carried out. In index patients with pathogenic variants, the evaluation of intraepidermal nerve fibre density in skin biopsy, corneal confocal microscopy and quantitative thermal threshold sensory testing (QST) were performed to evaluate possible small fibre neuropathy (SFN). Cerebrospinal fluid (CSF) analysis has been performed in patients exhibiting some features suggestive of neuroinflammatory disease, including Fabry-associated aseptic meningitis. Routine CSF examination is not a part of FD examination protocol in our Fabry disease centre.

#### 2.2.3. Cardiac Assessment

The cardiac evaluation consisted of detailed physical examination, resting 12-leads electrocardiography (ECG), echocardiography and cardiac MRI. Additionally, heart disease biomarkers, N-terminal prohormone of brain natriuretic peptide (NT-proBNP) and high-sensitive (hs) troponin I were analysed. 

#### 2.2.4. Nephrological Assessment

Serum creatinine and cystatin C were measured. An analysis of total protein in urine and urine albumin-to-creatinine ratio (ACR) were calculated. In patients with albuminuria or proteinuria, the albumin/protein excretion rate (AER, PER) was measured. Estimated glomerular filtration rate (eGFR, mL/min/1.73 m^2^) was calculated using the Chronic Kidney Disease Epidemiology Collaboration equation with serum creatinine and cystatin (CKD-EPIcreat, CKD-EPIcys formula) [15]. We assigned GFR and albuminuria categories according to Kidney Disease: Improving Global Outcomes (KDIGO) [16].

#### 2.2.5. Ophthalmological and Dermatological Assessment

Detailed ophthalmological assessment, including anterior segment examination in a slit lamp and fundoscopy and dermatological examination with an emphasis on the presence of angiokeratomas and capillaroscopy, was performed.

#### 2.2.6. α-Gal A Activity Assay

α-Gal A activities in plasma and leucocytes were measured by fluorometric method according to Mayes [17] as described previously [18]. Results obtained for patients’ samples were compared with enzyme activities in our group of FD hemizygotes and heterozygotes (for ranges, see Table 1). In some patients, enzyme activities were determined by the DBS method.

## 3. Results

The study included 23 individuals (16 index patients and 7 positively tested family members) carrying 8 different GLA gene variants. Pathogenic mutations were detected in 2 index patients and their 3 relatives, and VUS or likely benign variants in 14 index patients and their 4 relatives.

Eleven out of the sixteen index patients underwent a comprehensive examination for possible FD organ manifestations. Three index patients underwent incomplete (only cardiac and renal) assessments. Two index patients refused the examination.

Out of the seven positively tested relatives, six agreed to the complex clinical examination.

Stroke risk factors of index patients are shown in Table 2.

### 3.1. Pathogenic Mutation

A summary of FD characteristic symptoms and major organ involvement in individuals with pathogenic mutations is given in Table 1.

#### 3.1.1. Index Patients

**Figure 1:**
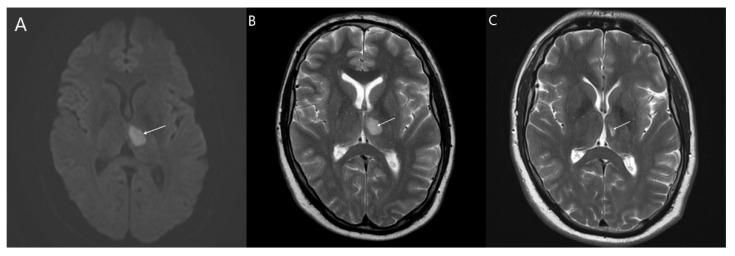
Axial MRI scans illustrate an infarct in the left medial thalamus (white arrows) of the patient 1.0 on day 3 post-stroke (**A**,**B**) and at 3 months post-stroke (**C**). (**A**) Diffusion-weighted imaging shows large lesion with restricted diffusion in the thalamus. (**B**) T2-weighted imaging shows hypersignal lesion; (**C**) After 3 months there is smaller hypersignal lesion as residual lesion.

**Figure 2:**
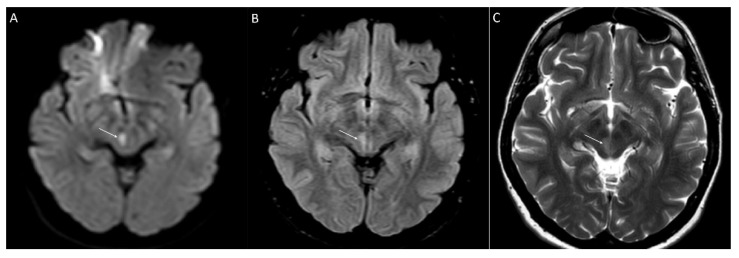
Axial MRI illustrates a lacunar infarct in the left midbrain of the patient 2.0 (white arrows) on day 3 post-stroke (**A**–**C**). (**A**) Diffusion-weighted imaging shows hypersignal lesion in midbrain (restricted diffusion). (**B)** Fluid-attenuated inversion recovery demonstrates hypersignal lesion. (**C**) T2-weighted imaging shows hypersignal lesion.

FD was diagnosed in two index individuals carrying pathogenic variants, a 34-year-old male (c.973G > A, G325S, patient 1.0.) and a 41-year-old female (c.1235_1236delCT, T412Sfs*?, patient 2.0.). Both variants were associated with the classical FD phenotype. The first presentation of FD in both probands was posterior circulation lacunar infarction (Figure 1 and Figure 2). Apart from lacunar infarcts, brain MRI did not provide evidence of any other pathology, not even WMLs. CSF analysis proved mild pleiocytosis in both. The cell count was 35 and 32 elements per mm^3^, respectively. A detailed examination of the CSF did not reveal the infectious cause of pleiocytosis. Oligoclonal bands were negative.

Further examination revealed the presence of FD-characteristic ocular findings. The renal and cardiac assessments did not reveal any pathology.

Although the man did not complain about any neuropathic sensory symptoms or pain, a further detailed examination of the peripheral nervous system (PNS) verified functional and structural abnormalities due to an isolated small fibre neuropathy. Skin biopsy revealed intraepidermal nerve fibre loss; confocal corneal microscopy demonstrated a decrease in corneal innervation, and abnormal QST confirmed a functional impairment of both A-delta and C fibres.

#### 3.1.2. Family Members

In the first family, FD was diagnosed in the proband’s 58-year-old mother (patient 1.1, G325S). She presented with skin and eye manifestations of FD. She did not suffer from neuropathic pain at present nor in the past. Major organ investigation revealed nonspecific brain WMLs (Fazekas 1).

In the second family, pathogenic mutations were detected in proband´s 12-year-old son and 10-year-old daughter (patients 2.1., 2.2., T412Sfs *?). The 12-year-old boy (patient 2.1.) presented with a typical triad of FD classical phenotype including ocular, skin and PNS manifestation: incipient cornea verticillata, angiokeratomas in typical localisation (periumbilical, buttocks and right thigh), painful episodic acroparesthesias and abdominal pain. Renal, heart and brain assessment did not show any abnormality. In his sister, a 10-year-old girl (patient 2.2.), we did not detect any abnormal finding apart from incipient cornea verticillata.

### 3.2. Variants of Unknown Significance and Likely Benign Variants

Assessment results of identified cases with VUS or likely benign variants (except D313Y) are depicted in Table 3. In one case, we identified a variant c.112 A > G, p.R38G. The patient, a 78-year-old woman (patient 5.0), presented with multiple angiokeratomas with a typical abdominal distribution. No other characteristic symptoms were found. Major organ examination showed mild renal impairment—PER of 0.19 g/24 h, slightly increased ACR of 3.74 g/mol and a mildly decreased eGFR 66 mL/min/1.73 m^2^ (CKD-EPIcreat), 75 mL/min/1.73 m^2^ (CKD-EPIcys). Cardiological examination revealed heart failure with preserved ejection fraction, atrial fibrillation, postcapillary pulmonary hypertension with mild right heart failure signs (echo signs of increased left ventricular filling pressures, elevated NT-proBNP—1926 ng/L); hs troponin was normal. Left ventricular hypertrophy (LVH) was not observed.

A previous stroke at the age of 40 was revealed in the patient’s medical history. The suspected cause was embolic caused by known atrial fibrillation with ineffective anticoagulation at the time of the index event.

The proband, a 47-year-old female (patient 3.0.), a variant c.352C > T, R118C carrier, had mild brain involvement—WMLs, mild proteinuria 0.20 g/L, PER 0.28 g/24 h with normal ACR and normal AER. Echocardiography detected a borderline LVH. No typical FD symptoms were revealed. Examination of her sister (52 years old, patient 3.1.) showed partially reduced α-GAL A activity, mild nonspecific brain and renal involvement (WMLs, eGFR CKD-EPIcys, 84.6 mL/min/1.73 m^2^). A 3-month episodic burning pain of her feet, that occurred in the context of a stressful personal situation, was revealed in the patient’s history.

In one case, a 70-year-old female patient (patient 4.0.), we identified a variant c.427G > A, A143T. There were no clinical signs or symptoms of possible FD, except for brain WMLs on MRI. In her 50-year-old daughter (patient 4.1.), we diagnosed a mild proteinuria 0.28 g/24 h, 0.14 g/L, ACR and AER were normal. The activities of α-GAL A in both leukocytes and plasma were overlapping with healthy controls values.

A male patient carrying the variant c.596T > C, V199A did not give his consent to further investigations due to his age and associated diseases (atrial fibrillation, kidney cancer, prostate cancer).

A total of eight individuals carrying c937G > T, D313Y variants underwent a clinical workup in specialised FD centre; two of them only agreed to a partial examination. Consent was not acquired in two individuals with index stroke (one refused investigation, one died). A daughter of a diseased 69-year-old female patient, who died of stroke complications, was found during family screening. Results are given in Table 3. Of note, none of the patients had relevant cardiac involvement, and minimal changes were observed in renal function in all but one patient (an 83 years-old female, patient 12.0) who refused further investigations.

### 3.3. Newly Identified Variant

A newly identified variant c.89G > A, p.R30K, was found in an 82-year-old woman (patient 6.0) with a lacunar posterior circulation ischaemic stroke. The patient herself refused further examination in an FD-specialised centre. Her 46-year-old daughter (patient 6.1) agreed to both genetic and clinical examinations, which revealed neither characteristic FD signs nor heart or brain pathology. However, renal investigation detected a slightly elevated AER 31.42 mg/24 h and ACR of 4.47 mg/mmol. Ophthalmological examination revealed very subtle signs of possible cornea verticillata. Enzyme activity was not reduced. Reconsidering the previously given consent and referring to the fact that there are no symptoms of the disease, she refused genetic testing and clinical examination of her children.

### 3.4. Further Management

After the evaluation of our cohort, we initiated therapy with migalastat in patients 1.0 and 1.1. As the variant T412Sfs*? is not amenable for chaperone therapy, patients 2.0 and 2.1. are receiving enzyme replacement therapy. All patients (including asymptomatic family members and VUS careers) remain under the care of the Czech FD centre, receiving a routine follow up for major organ involvement.

## 4. Discussion

FD in patients with cerebrovascular events has been assessed by different authors since 2005 [19]. However, detailed descriptions of phenotypic manifestations of detected variants are scarce in the literature. Since the initial misinterpretation of a genetic variant may impact a large group of patients, we carefully analysed organ manifestations of FD not only in known pathogenic mutations but in all variants detected in our screening. A total of 23 individuals with 8 different GLA variants were included in the study.

Both pathogenic mutations found (G325S, T412Sfs*?) have been previously described in the literature. The G325S mutation was associated with a nonclassical phenotype, while T412Sfs*? is known to cause the classical FD phenotype [20].

In G325S, only limited biochemical and clinical phenotype data were published. Lukas et al. provided biochemical phenotype characteristics of the mutated gene product, its pharmacological chaperone responsivity and described elevated lyso-Gb3, suggesting this mutation as pathogenic [21,22]. In a study by Kokotis et al. evaluating sweat gland innervation, a patient with the G325S variant was included. However, the phenotypic expression of the patient was not reported in detail [23]. The supporting evidence of the pathogenicity of the variant comes from another male patient with G325S variant visiting our FD centre, and not included in the cohort described in this paper. This 56-year-old gentleman suffers from severe multiorgan involvement (he underwent a kidney transplant, has severe LVH and had a transient ischaemic attack at the age of 49). His pre-treatment α-Gal A activity in leukocytes was severely depressed, reaching 1.1. nmol/mg/h (1.8% of mean value of our controls).

At the time of the diagnosis, our index patient with G325S mutation (patient 1.0.) showed biochemically low enzyme activity and high levels of lyso-Gb3. Detailed examination revealed a full spectrum of ocular pathology, small fibre neuropathy (although the patient did not report any sensory symptoms either at present or in his medical history, including childhood) and major organ complications. In our experience, it is not uncommon that FD patients do not report painful episodes (even in response to targeted questions) despite the clear presence of SFN on functional and structural examination methods. This may lead to a variant misclassification or to a delayed diagnosis. 

Based on the information obtained from a detailed examination of patients with the G325S variant, we concluded that the G325S mutation might result in a classical phenotype.

As compared to G325S, clinical descriptions of the T412Sfs*? phenotype in the literature are more frequent. The mutation is known to cause a classical phenotype. In our study, clinical symptoms of patient 2.0, together with typical painful crises that appeared in the patient’s 12-year-old son, confirm pathogenicity associated with the classic FD phenotype.

Interestingly, there was a mild pleocytosis discovered in CSF analysis in both index patients with the two pathogenic variants. CSF testing in patients with FD is not routinely performed. There have been reports in the literature describing “aseptic” or “chronic” meningitis in individual cases with FD suffering from neurological symptoms since 1985 [24]. Mild to moderate CSF pleocytosis corresponding to meningitis, either associated with headache and fever or not, have been documented. Simultaneous findings of lacunar infarctions predominantly in the posterior circulation, similar to in our study, have almost always been reported [24,25,26,27]. In contrast, in patients with ischaemic stroke without FD, pleocytosis is not commonly detected, although a very slight increase of white blood cells in the CSF may be seen [28]. Since aseptic meningitis is considered a rare phenotypic manifestation of FD, elevated cells in CSF may be a misleading feature when making the correct differential diagnosis against inflammatory diseases of the nervous system. However, as the findings in our patients show, after excluding inflammatory diseases, the association of a lacunar stroke with the CSF findings corresponding to aseptic meningitis in a young person should always lead to consideration of FD, whether CSF abnormalities are found in the early post-ischaemic period or detected later.

There is an ongoing debate about the pathogenicity and phenotypic manifestation of several VUS, including variants found in our cohort: R118C, A143T and D313Y.

The R118C variant was primarily identified in neonatal screening in 2004 [29]. Based on the biochemical characteristics of the enzyme, the later-onset FD phenotype was predicted [11]. Initially, this variant was considered pathogenic, leading to its inclusion in calculations of FD prevalence in young stroke patients screening [30]. Later, the variant was reclassified at VUS [29], although recently emerging information is in favour of the original classification [31].

The A143T variant was first described in 1997 by Eng et al. In their study, the variant was found in a 2-month-old child. The biochemical phenotype showed reduced α-GAL activity in the proband and his mother; clinical phenotype in the infant could not be evaluated, and his mother showed no signs of FD. Similarly, there was no history of FD in the family. The variant phenotypic expression was concluded by the authors as being unknown [32]. Later, Spada et al. assumed the A143T genotype leads to late-onset end-stage nephropathy [33]. Since then, the variant has been classified as a later-onset pathogenic mutation [11,34,35,36,37,38], including screening studies in stroke patients [39]. Biochemically, the variant carriers have been repeatedly shown to express decreased α-GAL A activity, both when assessed in vitro or/and in plasma or leucocytes analysis [11,34,35,40,41,42]. Based on the clinical information, the variant has been reclassified [40], and although it is still a matter of debate, it is currently referred to be VUS or a probably benign variant [12,43].

In our group, we identified three females (patients 3.0, 3.1, 4.0) with R118C and one with A143T (patient 4.1) variants. They presented with mild nonspecific symptoms (see Table 1), although in one case, we recorded a time-limited episode of paroxysmal neuropathic pain, similar to those seen in typical FD acroparesthesias. The activity of α-GAL A obtained, both in plasma and leukocytes, were lower as compared to our group of FD heterozygous women. Regrettably, we did not obtain consent to a detailed examination in R118C male relatives. In A143T, there were no living male members in the close family. In addition, patient 4.1. did not complete the investigation due to the COVID-19 pandemic.

Likewise, the clinical significance of variant D313Y has become a matter of controversy since it was first reported [44], especially after a duplicate missense GLA mutation has been detected in the same allele in a patient with a typical FD form [45,46]. However, a high residual D313Y enzyme activity and its dependence on pH (resulting in plasma pseudodeficiency [47]), normal values of lyso-Gb3 [48,49] and cases without clinical manifestations [47,49] indicate D313Y to be a benign variant.

Phenotyping in our cohort of patients with D313Y revealed symptoms attributable to FD in two individuals (Table 3). Patients 11.0 presented with cornea verticillata, and patient 13.1 suffered from neuropathic pain. After a detailed examination, patient 13.1. was diagnosed with coeliac disease. Thus, neuropathic pain might be explained by gluten-associated neuropathy [50]. In contrast, there was no clear explanation for cornea verticillata in the patient 11.0. Although some in vitro data attribute a potential risk to amlodipine which was taken by the patient [51], the risk of cornea verticillata with this drug was never reported.

In general, the clinical significance of some variants that most experts currently consider to be probably benign, especially R118C, A143T and D313Y, has been the subject of debate for years, and their association with FD still raises numerous questions. The lack of detectable lipid accumulation (negative biopsy of the kidneys, heart) strongly supports this classification. Nevertheless, a higher concentration of unmetabolised substrate, which does not yet lead to the formation of detectable inclusions, may already affect the balance of biochemical processes within the cell. Reduced enzyme activity, which is sufficient for metabolic processes under physiological conditions, may no longer be sufficient in situations with an increased demand on the metabolic glycosphingolipids turnover. Hypothetically, a partial and/or intermittent blockade of the catabolic pathway with subsequent accumulation of Gb3 can alter metabolites flow with an effect on other biochemical processes in the cell (for example, redistribution of glycosphingolipids in other subcellular districts) [52,53] without the formation of typical inclusions. Thus, hypothetically, there might be a possibility that transient, paroxysmal burning feet pain in patients 3.1 with the R118C variant during periods of severe stress might be associated with the GLA variant. Moreover, it should be noted that the determination of α-GAL activity was performed in plasma and leukocytes in vitro. In vivo catalytic α-GAL A activity may be different and may show cell-to-cell variability [54].

Many other genetical–biochemical–environmental interactions may then contribute to the fact that a benign variant in a particular individual may, under certain circumstances, act as a risk factor contributing to organ manifestation (e.g., cerebrovascular events). On the other hand, in some cases, storage has been described in VUS carriers [31,55]. Multilayer inclusions may be caused by drug-induced phospholipidosis (DIP). Some preparations are known to lead to lysosomal deposition and to clinical manifestations resembling symptoms of FD, e.g., cornea verticillate [56,57]. Amiodarone and aminoquinolines are known to cause cornea verticillata by means of DIP. Although the DIP potential has also been demonstrated in commonly used carvedilol and amlodipine in vitro, their association with cornea verticillata is not known [51]. Nevertheless, a precise medical history is essential also in individuals who have been found to be carriers of the variant in the GLA gene and suffer from cornea verticillate [58]. In our group, all patients with pathological mutations, including children, presented with the cornea verticillata. Among other individuals, patient 6.1 (R30K) presented with very subtle findings, requiring additional eye monitoring.

Some limitations of the study should be noted. The different screening methods were used for males and females in stroke patients. The GLA gene sequencing was performed only in males with abnormal values of α-Gal-A activity and/or elevated Lyso-Gb3. In contrast, all samples obtained from female patients were sequenced. This may lead to underestimated frequency of several variants of unknown significance and benign variants in hemizygous males in whom a high residual enzyme activity is preserved and lyso-Gb3 remains low. Future studies aiming to detect the whole spectrum of genetic variants should also use GLA sequencing in male patients.

## 5. Conclusions

In conclusion, we present a descriptive clinical study including 23 patients (16 suffering from stroke) with 8 different GLA variants. We propose to reclassify the pathogenic variant G325S from causing late-onset FD type to a variant resulting in the classical one. The newly identified GLA variant R30K could not be accurately assessed for pathogenicity. The combination of a lacunar stroke in a young person with the CSF finding of aseptic meningitis is highly suggestive of FD.

Taking into account the availability of specific FD treatment, it is of great importance to share patients’ data. In order to better characterise VUS, not only probands but also all asymptomatic variant carriers diagnosed by family screening should be followed and evaluated prospectively.

These data, in the future, will help to distinguish symptoms attributable to FD from nonspecific comorbidities in benign GLA variants carriers.

## Figures and Tables

**Table 1:** Genotype and phenotype characteristics of patients with pathogenic mutation and variants of unknown significance in GLA gene.

Nr.	Variant	Demographic Information			Biochemical Phenotype				Clinical Phenotype													
	AA Change	Sex	BMI	Age (years)	α-Gal A Activity			Lyso-Gb_3_	Characteristic Symptoms							Major Organ Involvement						
									Eyes			Skin	Nervous System					Heart			Kidney ^†^	
													PNS			CNS						
					DBS nmol/mL/h	Plasma nmol/mL/h	Leukocytes nmol/mg/h	DBS ng/mL	Tortuous vessels	Cornea verticillata	Fabry cataract	AG	Sweating	Neuropathic pain or SFN	GIT	Stroke	WML/FS	ECG	LVH	LGE	Alb	GFR
**FD pathogenic GLA mutation**																						
1.0	G325S	M	27.7	34	<2.8	0.22 ^#^	11.3 ^#^	19.8	+	+	+	-	-	+	-	+	-/0	-	-	-	A1	G1
1.1	G325S	F	28.4	58	na	na	na	3.6	+	+	-	+	-	-	-	-	+/1	-	-	-	A1	G2
2.0	T412Sfs*?	F	26.9	41	na	6.22	33.1	5.5	+	+	-	-	-	-	-	+	-/0	-	-	-	A1	G1
2.1	T412Sfs*?	M	18.6	12	na	0.18	0.1	26.9	-	+	-	+	-	-	+	-	na	-	-	na	A1	G1
2.2	T412Sfs*?	F	20.9	10	na	1.62	26.1	na	-	+	-	-	-	-	-	-	na	-	-	na	A1	G1
**GLA variant of unclear significance or probably benign variant**																						
3.0	R118C	F	30.8	47	na	0.26	64.7	0.8	-	-	-	-	-	-	-	+	+/1	-	+	-	A1	G1
3.1	R118C	F	25.7	52	na	2.64	34.5	1.0	-	-	-	-	-	+	-	-	+/1	-	-	na	A1	G2
4.0	A143T	F	25.2	70	na	1.88	30.1	1.1	-	-	-	-	-	-	-	+	+/1	LAH	-	-	A1	G1
4.1	A143T	F	31.2	50	na	na	na	1.2	Na	na	na	-	-	-	-	-	na	-	na	na	A1	G1
5.0	R38G	F	24.7	78	na	1.7	22.7	1.0	-	-	-	+	-	-	-	+	+/2	AF	-	na	A2	G2
6.0	R30K	F	na	83	na	na	na	1.0	Na	na	na	na	na	na	na	+	na	-	na	na	na	na
6.1	R30K	F	22.9	46	na	3.74	68.5	1.0	+	-	-	-	-	-	-	-	-/0	-	-	-	A1	G1
7.0	V199A	M	na	78	9.4	na	na	1.3	Na	na	na	na	na	na	na	+	na	AF	na	na	na	na

AA = amino acid; α-GAL A = Alpha-galactosidase A; Nr. = patient number; BMI = body mass index; y = years at the time of examination in the Fabry disease centre, PNS = peripheral nervous system; CNS = central nervous system; DBS = dry blood spot; AG = angiokeratomas; SFN = small fibre neuropathy; GIT = gastrointestinal symptoms; WML = white matter lesions; FS = Fazekas scale; ECG = electrocardiography; LVE = left ventricular enlargement; LGE = late gadolinium enhancement; GFR = glomerular filtration rate; M = male; F = female; LAH = left anterior hemiblock; AF = atrial fibrillation; + indicates the presence of a finding; - indicates the absence of a finding; na = not available; Alb = albuminuria; ^†^ category graded by kidney disease improving global outcomes [16]; plus + indicates organ involvement; minus - indicates no pathology. Controls: α-Gal A in leukocytes 25–103 nmol/mg/h, mean value ± SD 59.7 ± 14.6 nmol/mg/h, n = 477, in plasma 2.4–19.4 nmol/mL/h, mean value ± SD 6.1 ± 2.8 nmol/mL/h, n = 322, in DBS 15.3 μmol/L/h; lysoGb3 in DBS 1.8 ng/mL. ^#^ Enzyme activity assessment while taking migalastat, pre-treatment activity available from DBS.

**Table 2:** The major risk factors for ischaemic stroke in index patients.

	Mutation Characteristics		Nonmodifiable Risk Factors		Modifiable Risk Factors					
Nr.	c.DNA Change	Amino Acid Change	Sex	Age (years)	Hyper Tension	Diabetes	Heart Disease	Dyslipidemia	Previous Stroke or TIA	Smoking
**Pathological GLA variants**										
1.0	c.973G > A	p.Gly325Ser	M	34	-	-	-	-	-	+
2.0	c.1235_1236delCT	p.Thr412Serfs*?	F	40	-	-	-	-	-	+
**GLA variants of unknown significance or probably benign**										
3.0	c.352C > T	p.Arg118Cys	F	47	+	-	-	-	-	-
4.0	c.427G > A	p.Ala143Thr	F	70	+	-	-	+	-	-
5.0	c.112A > G	p.Arg38Lys	F	78	+	-	AF	-	+	-
6.0	c.89G > A	p.Arg30Lys	F	83	+	-	-	-	-	-
7.0	c.596T > C	p.Val199Ala	M	78	-	-	IHD, AF	-	-	+
8.0 #	c.937G > T	p.Asp313Tyr	M	41	+	-	-	-	-	-
9.0	c.937G > T	p.Asp313Tyr	M	54	-	-	PFO	+	-	+
10.0	c.937G > T	p.Asp313Tyr	F	44	-	-	-	-	-	-
11.0	c.937G > T	p.Asp313Tyr	M	64	+	-	IHD	+	-	-
12.0	c.937G > T	p.Asp313Tyr	F	83	+	+	IHD	+	-	-
13.0	c.937G > T	p.Asp313Tyr	F	72	+	-	IHD	-	-	-
14.0	c.937G > T	p.Asp313Tyr	F	71	+	-	PFO	+	-	-
15.0	c.937G > T	p.Asp313Tyr	M	59	+	-	-	-	-	+
16.0	c.937G > T	p.Asp313Tyr	F	44	-	-	-	-	+	-

Nr. = patient number; y = years, at the time of cerebrovascular event; M = male; F = female; TIA = transient ischaemic attack; AF = atrial fibrillation; IHD = ischaemic heart disease; PFO = patent foramen ovale; + indicates the presence of a finding; - indicates the absence of a finding; # the patient with basal ganglia haemorrhage.

**Table 3:** Phenotype characteristics of patients with variant c.937G > T, p.Asp 313Tyr (D313Y).

Nr.	Demographic Information			Phenotype															
	Sex	BMI	Age (years)	Lyso-Gb_3_ (ng/m)	Characteristic Symptoms							Major Organ Involvement							
					Eyes			Skin	Nervous System						Heart			Kidney ^†^	
									PNS			CNS			ECG	LVH	LGE	alb	GFR
					Tortuous Vessels	Cornea Verticillata	Fabry Cataract	AG	Sweating	Neuropathic Pain	GIT	Stroke	WML/FS	Other					
8.0	M	25.9	43	1.6	+	-	-	-	-	-	-	+	+/2	-	LAH	-	-	A1	G2
9.0	M	26.7	54	1.0	-	-	-	-	-	-	-	+	+/1	-	-	-	na	A2	G1
10.0	F	35.9	44	1.0	+	-	-	-	-	-	-	+	-/0	MSA	-	-	na	A1	G2
11.0	M	25.8	64	1.1	+	+	-	-	-	-	-	+	+/2	MSA, MCA stenosis	iRBBB	-	na	A1	G1
12.0	F	n.d.	83	1.4	na	na	na	-	-	-	-	+	na	-	-	-	na	na	G3b
13.0	F	n.d.	72	0.9	na	-	na	na	na	na	na	+	na	na	na	na	na	na	na
13.1	F	36.9	47	1.1	-	-	-	-	-	+	-	-	-/0	-	-	-	na	A1	G1
14.0	F	31.9	71	1.2	-	-	-	-	-	-	-	+	-/0	epilepsy	-	-	na	A1	G1
15.0	M	38.4	59	1.1	na	na	na	na	na	na	na	+	na	na	na	-	na	na	G1
16.0	F	17.4	44	1.1	na	na	na	-	-	-	-	+	na	-	na	na	na	na	G1

Nr. = patient number; BMI = body mass index; y = years at the time of examination in the Fabry disease centre; PNS = peripheral nervous system; CNS = central nervous system; AG = angiokeratomas; GIT = gastrointestinal symptoms; WML = white matter lesions; FS = Fazekas scale; MSA = mild sleep apnea; MCA = middle cerebral artery; ECG = electrocardiography; LVE = left ventricular enlargement; LGE = late gadolinium enhancement; GFR = glomerular filtration rate; M = male; F = female; LAH = left anterior hemiblock; iRBBB = incomplete right bundle branch block; na = not available; alb = albuminuria; ^†^ category graded by kidney disease improving global outcomes [16]; plus + indicates organ involvement; minus - indicates no pathology.

## Data Availability

The data supporting reported results of this study are available upon reasonable request to the corresponding author.
